# Development of a Real-Time Dashboard for Overdose Touchpoints: User-Centered Design Approach

**DOI:** 10.2196/57239

**Published:** 2024-06-11

**Authors:** Amey Salvi, Logan A Gillenwater, Brandon P Cockrum, Sarah E Wiehe, Kaitlyn Christian, John Cayton, Timothy Bailey, Katherine Schwartz, Allyson L Dir, Bradley Ray, Matthew C Aalsma, Khairi Reda

**Affiliations:** 1 School of Informatics, Computing, and Engineering Indiana University Indianapolis Indianapolis, IN United States; 2 Children’s Health Services Research Department of Pediatrics Indiana University School of Medicine Indianapolis, IN United States; 3 Indiana Clinical and Translational Science Institute Indianapolis, IN United States; 4 Indiana Management Performance Hub Indianapolis, IN United States; 5 Department of Psychiatry Indiana University School of Medicine Indianapolis, IN United States; 6 RTI International Research Triangle Park, NC United States

**Keywords:** overdose prevention, dashboards, fatality review, data integration, visualizations, visualization, dashboard, fatality, death, overdose, overdoses, overdosing, prevention, develop, development, design, interview, interviews, focus group, focus groups, touchpoints, touchpoint, substance abuse, drug abuse

## Abstract

**Background:**

Overdose Fatality Review (OFR) is an important public health tool for shaping overdose prevention strategies in communities. However, OFR teams review only a few cases at a time, which typically represent a small fraction of the total fatalities in their jurisdiction. Such limited review could result in a partial understanding of local overdose patterns, leading to policy recommendations that do not fully address the broader community needs.

**Objective:**

This study explored the potential to enhance conventional OFRs with a data dashboard, incorporating visualizations of touchpoints—events that precede overdoses—to highlight prevention opportunities.

**Methods:**

We conducted 2 focus groups and a survey of OFR experts to characterize their information needs and design a real-time dashboard that tracks and measures decedents’ past interactions with services in Indiana. Experts (N=27) were engaged, yielding insights on essential data features to incorporate and providing feedback to guide the development of visualizations.

**Results:**

The findings highlighted the importance of showing decedents’ interactions with health services (emergency medical services) and the justice system (incarcerations). Emphasis was also placed on maintaining decedent anonymity, particularly in small communities, and the need for training OFR members in data interpretation. The developed dashboard summarizes key touchpoint metrics, including prevalence, interaction frequency, and time intervals between touchpoints and overdoses, with data viewable at the county and state levels. In an initial evaluation, the dashboard was well received for its comprehensive data coverage and its potential for enhancing OFR recommendations and case selection.

**Conclusions:**

The Indiana touchpoints dashboard is the first to display real-time visualizations that link administrative and overdose mortality data across the state. This resource equips local health officials and OFRs with timely, quantitative, and spatiotemporal insights into overdose risk factors in their communities, facilitating data-driven interventions and policy changes. However, fully integrating the dashboard into OFR practices will likely require training teams in data interpretation and decision-making.

## Introduction

### Background

The escalating drug overdose epidemic in the United States continues to pose a major public health challenge. Previous research has identified general risk factors that are linked to increased overdose rates [1-3], including unstable housing [4,5], recent release from incarceration [6,7], and frequent visits to the emergency department (ED) [8-11]. However, overdose risk factors exhibit considerable variation across communities and are influenced heavily by geographic and demographic disparities, particularly in access to health care and prevention services [9,12]. Moreover, the evolving nature of the epidemic has led to shifting risk profiles among different subpopulations [13]. These disparities underscore the need for timely and data-driven interventions that are tailored to the specific needs and challenges of local communities.

One mechanism for implementing targeted, community-specific interventions is through local Overdose Fatality Reviews (OFRs). Modeled after child fatality reviews [14,15], OFR teams comprise reviewers from multiple agencies who conduct collaborative, in-depth reviews of case files for individuals who have died of overdose [16,17]. Through these detailed case reviews, OFRs identify service gaps and recommend strategies to prevent future overdoses in their communities. The use of OFRs has gained momentum, with teams operating across various US localities [18]. However, current OFR practices primarily focus on reviewing only a handful of cases, typically 2 to 5 monthly or quarterly [19]. These cases typically represent a small fraction of the total fatalities occurring in their jurisdiction. While informative, the emphasis on a few individual cases could skew the review process, leading to OFRs making recommendations that do not fully address broader overdose trends.

As local governments continue to collect data on overdose events, there is an opportunity to leverage these data to enhance the OFR process. Previous research demonstrates the value of linking administrative data sets routinely collected by state governments (eg, calls to emergency services and incarceration records) with overdose mortality data [20-24]. For example, cross-referencing the records of decedents who experienced overdoses from across various data sets allows for uncovering their “touchpoints”—interactions with health and social services and other local systems they had before their overdose. When brought to light, touchpoints offer key opportunities to engage at-risk individuals and connect them with prevention services and treatments [25-27]. Analyses to identify touchpoints have so far been performed manually by researchers. However, the process is amenable to automation, enabling continuous assessment of touchpoint characteristics. The results can then be communicated in real time to local OFRs through a dashboard, providing review teams with up-to-date, quantitative information on the trajectories of decedents in their communities.

Dashboards have proven invaluable in public health settings [28,29] owing to their ability to visually summarize key metrics and statistics [30,31], thereby aiding surveillance and fostering evidence-based responses to emerging health threats [32,33]. Furthermore, dashboards are conducive to collaborative sense making among multiple individuals [34-36]. This feature makes them particularly suited to fatality review meetings, which are designed to be collaborative and deliberative in nature. Numerous dashboards have been developed to visualize drug overdose–related data [37-39]. However, existing solutions are primarily intended to surveil the level and distribution of overdoses as opposed to understanding events that *precede* them. Few of the earlier dashboards showcase touchpoints at the local level or update data in real time, making them less suited for understanding system-level gaps or for deriving prevention-oriented insights.

### Aims

This study presents findings from human-centered research, design, development, and initial evaluation of a dashboard aimed at supporting OFR teams by visualizing overdose touchpoint statistics. The objective was to provide county-level OFR teams with timely and actionable data on events that consistently *precede* fatal overdoses in their communities. In doing so, we aimed to illuminate additional opportunities for interventions at the population level beyond what can be gleaned from individual fatality case reviews. The goal was to increase the chance of successful targeting and implementation of OFR recommendations. This stands to improve overdose prevention and reduce the number of preventable deaths.

## Methods

### Overview

To design a dashboard suitable for the needs of OFRs, we adopted a user-centered design framework [40,41] drawing on participatory methods to engage stakeholders in the process [42,43]. Specifically, we conducted focus groups with a panel of OFR experts to elicit perspectives on requirements and data needs, envision design possibilities, and document potential challenges. The elicited requirements were then used to develop exploratory visualizations of touchpoints data. The initial visualizations were further refined based on feedback from the expert panel. Subsequently, the revised visualizations were used to develop a web-based dashboard that is hosted by the Indiana state government.

### Study Setting and Data Sources

We partnered with the state government of Indiana to prototype and develop the sought touchpoints dashboard. Indiana has a nationally recognized role in organizing and convening OFRs, with 28 active review teams organized at the county level and supported by the Indiana Department of Health. Similar to many other states, Indiana maintains a comprehensive and up-to-date database of fatal overdoses. This database includes all suspected accidental poisonings (coded as X40-X44), intentional poisonings (X60-X64), assaults by drug (X85), and cases of undetermined intent (Y10-Y14) that occurred among Indiana residents. In addition to overdose data, the state maintains administrative data sets from various agencies, including incarceration records, emergency and medical service use, and prescription dispensation. Importantly, these administrative data sets are linkable to the overdose mortality records. The Indiana Management Performance Hub (MPH), a state-level agency, serves as a central repository for these data sets, which are gathered from the corresponding agencies.

To identify events that precede drug-related fatalities, overdose cases are linked to administrative data sets at the individual level. This linking procedure is performed by the MPH using a probabilistic matching algorithm that considers identifiers such as the decedent’s name, date of birth, and social security number, among others. This process allows for the reconstruction of past interactions with various touchpoints for each identifiable decedent. Subsequently, deidentified statistics about these interactions are pushed to the dashboard for visualization. This linkage process is performed weekly, enabling (near) real-time updates of the visualizations.

### User-Centered Design Process

To inform the design of the dashboard, we conducted 2 focus groups with a panel of OFR experts. We recruited participants via email, inviting experienced OFR practitioners and early developers from across the United States. Our goal in these focus groups was to understand OFR information needs and leverage the panel’s experience in conceptualizing, co-designing, and refining visualizations. The focus groups took place virtually using Zoom videoconferencing software (Zoom Video Communications). A virtual whiteboard was used to place and arrange “Post-it”–style notes. Participating experts were recruited from the same pool, with later focus groups involving fewer participants to allow for convergence and facilitate more in-depth feedback. The focus groups were video recorded, transcribed, and analyzed using thematic analysis techniques [44].

The first focus group sought to uncover data access barriers and needs for OFR teams. A total of 13 experts participated in the discussion. Participants were first prompted to share challenges and “pain points” regarding access to data. In a second activity, participants were divided into 2 breakout groups to identify key data attributes essential for review teams. They also gave high-level design parameters for the dashboard. Finally, participants reflected on their hopes and concerns for the dashboard’s integration into OFR processes, emphasizing potential positive outcomes and addressing apprehensions.

On the basis of the findings of the initial focus group, we created a series of 6 initial visualizations that illustrate overdose touchpoints using a static snapshot of the MPH-linked data set described previously. These initial visualizations served as the foundation for a second focus group with the participation of 6 experts. During this session, a facilitator presented each of the 6 visualizations and prompted participants for feedback. Specifically, participants were asked to evaluate the ease of understanding of these visualizations and their potential usefulness in the OFR process. We sought additional input by conducting a survey of 5 experts. The survey presented the same initial visualizations and requested open-ended comments on their intuitiveness and utility. Insights gathered from the survey along with feedback obtained during the second focus group were used to refine the visualizations and develop an interactive dashboard.

### Dashboard Evaluation

To obtain feedback on the final dashboard, we conducted an initial assessment with 3 OFR experts. Participants were asked to perform a series of data extraction tasks (eg, identifying the touchpoint with the highest prevalence). In addition, they were prompted to make recommendations based on the observed touchpoint patterns, simulating the use of the dashboard within a typical OFR meeting.

### Ethical Considerations

This human-centered research was reviewed and approved by the Indiana University institutional review board (approval 17809). Participants received an information sheet explaining the study goals and procedures before agreeing to take part. The analysis of state mortality and administrative data sets, while not considered human participant research, followed state legal and ethical procedures. The dashboard displays only aggregate, population-level visualizations. No individual records are released or displayed to preserve anonymity. Furthermore, special care was taken to minimize the risk of reidentification by withholding actual event counts and substituting with percentages. Participants received a US $100 gift card as compensation.

## Results

### Overview

Participants highlighted barriers faced by OFRs in accessing and interpreting data within the context of fatality reviews. They also provided insights on what data attributes and features would be most useful for OFRs to look at. We report these findings and discuss how we incorporated them to create a real-time dashboard for visualizing overdose touchpoints.

### Barriers to Accessing and Using Data

#### Data Accessibility

Several participants highlighted the lack of access to data as one of the major barriers in fatality reviews. Some of these barriers stem from challenges in sharing available data due to legal restrictions, data security, and privacy concerns:

Asking our state offices for data would result in, “Sorry, we can't share on the state level.” There [needs to] be intergovernmental agreements between state police or our mental health or our human services or our health department.

P2

[Gaining access] is always an issue, and especially without laws that allow for the OFRs to get this. I know we had a lot of laws related to the child death review teams that I worked with that allowed us access to data, but it wasn't always the same for other death review teams.

P11

While recognizing existing regulatory and logistical obstacles, participants anticipated that increasing data access could empower OFRs to make more informed decisions:

We’re trying to drive positive change that could maybe be implemented statewide, and they just give us a little bit. It [data] would give us the power to make better decisions.

P2

In addition to data access, the quality and accuracy of the data were also brought up as a prominent issue for OFRs, especially because of acknowledged variations in how data are coded and measured across different organizations. For example, 1 participant cited different standards for classifying services, noting that such inconsistencies could lead to misinterpretation:

When it’s really law enforcement heavy, they’re not understanding the public health ramifications of criminal justice involvement. It affects the lens from which data’s being collected. So, when I go through the qualitative data...we’ve got people identifying jail substance use services as harm reduction, [and] you end up collecting some inaccurate data, which then misinforms the big picture.

P7

#### Influx of Case-Specific Data

While obtaining population-level data in certain arenas proved challenging, another concern was the vast amount of case-specific data that OFRs must already contend with. Participants noted that review teams are increasingly tasked with handling large volumes of individual reports from multiple systems, which often need to be manually and qualitatively analyzed at considerable time and effort:

OFRs collect an enormous amount of data, but you really need a whole army of researchers to be able to analyze it, especially the qualitative data. When the teams are putting forth all of these recommendations, it’s just so hard to go through all the information and make a meaningful plan of it.

P7

Extensive data on individual death circumstances (as opposed to population-level statistics) reflect a conventional OFR focus on in-depth reviews of a few strategically selected cases. However, with the sheer number of overdose fatalities, it becomes difficult for OFRs to ensure that the selected cases represent the broader overdose patterns and risk factors prevalent in their community. One participant put it as follows:

[My experience] is that they would just randomly pick cases and then do a really deep dive into those cases, but you have no way to actually ensure that those are representative...And so, my hope had been that we would have certain [data] fields that we could have someone enter, and then that would allow us to do really large-scale analysis over the course of multiple years...[This] would have allowed us to really have a good sense as it relates to a variety of factors, but there just wasn’t capacity. So, then we're just picking cases that look good or meet some theme to be able to have a more robust conversation at any given meeting. But again, they're not necessarily representative and you don't end up having the whole picture.

P18

### Key Data Types and Attributes for the Dashboard

Participants identified key data attributes that they deemed essential for inclusion in a dashboard. We divided these attributes into 3 categories: touchpoints, social determinants of overdose risk, and case-specific data.

#### Touchpoints

Touchpoints represent interactions with systems and services before overdose. Thus, they serve as opportunities to connect people who use drugs with additional prevention services and treatments, potentially mitigating the risk of future overdoses. A frequently recurring set of touchpoints identified by experts was interaction with the justice system. For instance, the duration between a decedent’s overdose and their last incarceration or residential treatment was cited as particularly important:

Were they justice involved or not at any point, but also the average distance in time from their last incarceration...So, to see were they in that window of high risk. And same if they were in residential treatments as average number of days.

P3

Average days out from treatment and incarceration because I feel like those are solid spaces that action can be taken.

P5

Several participants pointed to interactions with justice systems broadly as key touchpoints. Agencies such as county sheriffs, local police departments, and child protective services were thought to play a crucial role in an individual’s risk of overdose both positively and negatively:

Justice systems can either be a force of treatment or a barrier to treatment. I think that involvement is really important...the extent of involvement can be really helpful to inform the justice system and the legislative changes that could help.

P4

Participants noted that data on criminal justice touchpoints might reveal new prevention opportunities or support policy recommendations, such as facilitating continued treatment for institutionalized individuals:

...keep people engaged in treatment, [such that] we’re not disrupting treatment by violating [ie, rearresting] people and incarcerating them...It’s a fruitful area for policy change. Most of our policy changes and recommendations from our OFR have been in the justice space.

P3

In addition to justice systems, participants noted interactions with health and medical facilities as crucial touchpoints. This included visits to the ED and emergency medical services (EMS):

Do we have one [attribute] here [on] the last date of medical intervention? Maybe like an ED visit or anything like that?

P4

There’s an ED and EMS interaction right at the center there.

P5

Overall, three primary touchpoint categories emerged: (1) encounters with the justice system, such as incarceration; (2) engagement with health services, including ED and EMS interactions; and (3) involvement with residential treatment services. These touchpoints were recognized by participants as crucial opportunities for understanding risk factors and implementing services to close treatment gaps. Importantly, participants emphasized the typical interval between these touchpoints and overdose events as a critical feature to emphasize in the dashboard.

#### Social Determinants of Health

A second set of data attributes identified pertained to the social condition of the individuals themselves, which could shed light on factors that contribute to elevated overdose risk. For example, one of these factors was demographics:

Basic demographic information like poverty level, education level, homelessness. Anything that would affect those social determinants of health.

P4

A second factor was individuals’ access to harm reduction services, as the same participant noted:

I was going to add...access to harm reduction services. So, what an environmental scan of resources or access to naloxone, treatment centers, syringe service programs, all those different community level access points.

P4

A third factor was housing, encompassing the shelter system and housing agencies:

Access to housing. Or maybe it’s access to shelter because it could be both. There’s housing policy, but then there’s also the shelter systems.

P5

A fourth factor was the availability of transportation, which, according to participants, could influence an individual’s access to treatment and harm reduction services:

Transportation between places: how easy is it for someone to get from point A to point B? Even if there’s a syringe service program down the street, can they get to it? That kind of thing.

P5

Finally, participants also identified upstream social determinants such adverse childhood experiences as potentially relevant factors in assessing overdose risk:

...and some of that I think would fall under ACEs too because even if they’re an adult, finding out if they were involved in that system as a child, trying to make some of those associations maybe.

P5

#### Case-Specific Data

Alongside touchpoints and social determinants of health, participants cited certain case-specific data, including toxicology reports, interviews with next of kin, and the decedent’s circumstances at the time of death (eg, their position and whether they were alone). While these attributes are relevant to reviewing individual cases, they were not considered for inclusion in the dashboard as our primary objective was to offer population-level data that complement rather than supplant the conventional OFR case review model.

### Apprehensions and Foreseen Challenges

Although participants were positive about the potential of the dashboard to enhance the OFR process, there were a few apprehensions. A major concern was the risk of unintentional identification of decedents in smaller counties, where there are fewer overdose deaths:

I’ve been aware of a couple different cases in relatively small communities where all the data says one thing, and of course, as a small community, we know exactly who we’re talking about.

P15

I think one [concern] would be that the information might be too identifiable, especially for small communities.

P8

Participants discussed the ethics of displaying data that might be inaccurate or that could be misused (eg, by law enforcement) to target at-risk individuals:

...that it has inaccurate and bad data. And that it is used for evil rather than for good...That it’s not used for bad downstream consequences kind of thing.

P6

Finally, participants raised the risk of misinterpreting data, noting that, while OFRs have expertise in studying individual histories of decedents to formulate recommendations, they are less familiar with analyzing population-level statistics. Some voiced reservations about OFR teams’ data literacy and their ability to draw appropriate inferences from such quantitative data. For instance, 1 participant gave an example of how a decrease in emergency medical events could be erroneously interpreted as a reduction in overdoses when it might only reflect fewer 911 calls:

That [error] where you have a number and you think it means one thing, but it means another thing...You have measured something, but not the thing that you are taking that thing to be.

P1

Others commented on the potential downstream consequences of misinterpreting data, which could manifest as inappropriate or even detrimental recommendations:

We’ve seen this trend in our data. That probably means X, Y, Z. And you might be right. You might be very wrong, and the data might be used to justify a policy or programmatic intervention that could in fact exacerbate it.

P17

Helping users interpret data accurately was deemed by participants as a critical consideration for the dashboard. Equally important was not to inundate OFRs with even more (population-level) data that teams may lack the bandwidth or data literacy skills to act upon. These insights underscore the need to craft intuitive data visualizations that can be comprehended accurately with minimal effort. Moreover, such displays should actively guide OFR teams into making valid inferences from the data presented.

### Touchpoints Selection

Our observations point to a longstanding limitation of current OFR practices, which focus on reviewing a handful of overdose cases at every meeting. OFR experts appeared to recognize the shortcomings of this model when pitted against the sheer volume of overdoses. Simultaneously, participants expressed strong interest in accessing additional data sets that would paint a broader picture of overdose risk factors and touchpoints in their community, provided that these data were consistently coded, intuitively summarized, and presented in a manner that did not overburden review teams.

Among the data emphasized by participants, touchpoints emerged as particularly actionable as they represent system interactions preceding overdose events. For instance, the proportion of decedents who used various touchpoints offers predictive power to identify the most effective points within the system for targeting at-risk individuals with prevention services. Moreover, understanding the typical time window between a touchpoint and an overdose event, along with the frequency of touchpoint use, can assist in designing interventions, including their timing and regularity.

Drawing on the insights of the expert panel and data availability in Indiana, we incorporated 5 touchpoint types into the dashboard: jail bookings, prison releases, visits to the ED, encounters with EMS, and prescriptions for controlled substances (eg, opioid analgesics). We excluded ED and EMS encounters occurring within a 24-hour window of death as those are likely to represent interactions directly related to the overdose event as opposed to potential touchpoints for prevention purposes. Interactions with both justice and medical systems were identified as key by the expert panel. Prescriptions for scheduled drugs, such as opioid analgesics, were included as touchpoints due to their established association with overdose risk [25]. We also included the dispensation of buprenorphine prescriptions as a touchpoint in the initial dashboard design. However, concerns were raised that singling medication for opioid use disorder as a separate touchpoint could cause it to be misconstrued as a causal risk factor for overdose. Consequently, buprenorphine data were merged and included among the general prescription dispensation touchpoint for scheduled drugs. Table 1 provides a summary of these touchpoints as highlighted by participants and featured in the dashboard. Although interactions with residential treatment services were identified as an important touchpoint by participants, related data are not centrally tracked by the state and, hence, were not available for inclusion in the dashboard. Moreover, social determinants of health are not currently included despite their relevance as the dashboard was intended to prioritize opportunities for immediate as opposed to upstream prevention. Case-specific attributes were also not considered for inclusion because they would be redundant to the traditional OFR case review process.

**Table 1:** Data types and attributes as identified by experts and featured in the dashboard.

Data type and attribute		Identified by expert panel?	Included in dashboard?
**Touchpoint**			
	Jail booking	No	Yes
	Release from prison	Yes	Yes
	Visit to the ED^a^	Yes	Yes
	Encounter with EMS^b^	Yes	Yes
	Interaction with residential treatment services	Yes	No
	Prescription dispensation for scheduled drugs, including opioid analgesics and MOUD^c^	No	Yes
**Social determinants**			
	Demographics	Yes	No
	Educational level	Yes	No
	Poverty	Yes	No
	Access to harm reduction services	Yes	No
	Housing	Yes	No
	Access to transportation	Yes	No
	Adverse childhood experiences	Yes	No
**Case-specific attributes**			
	Toxicology report	Yes	No
	Next-of-kin interviews	Yes	No
	Circumstances of death (eg, body position and presence of witnesses)	Yes	No

^a^ED: emergency department.

^b^EMS: emergency medical services.

^c^MOUD: medication for opioid use disorder.

### Initial Visualization Attempts

**Figure 1:**
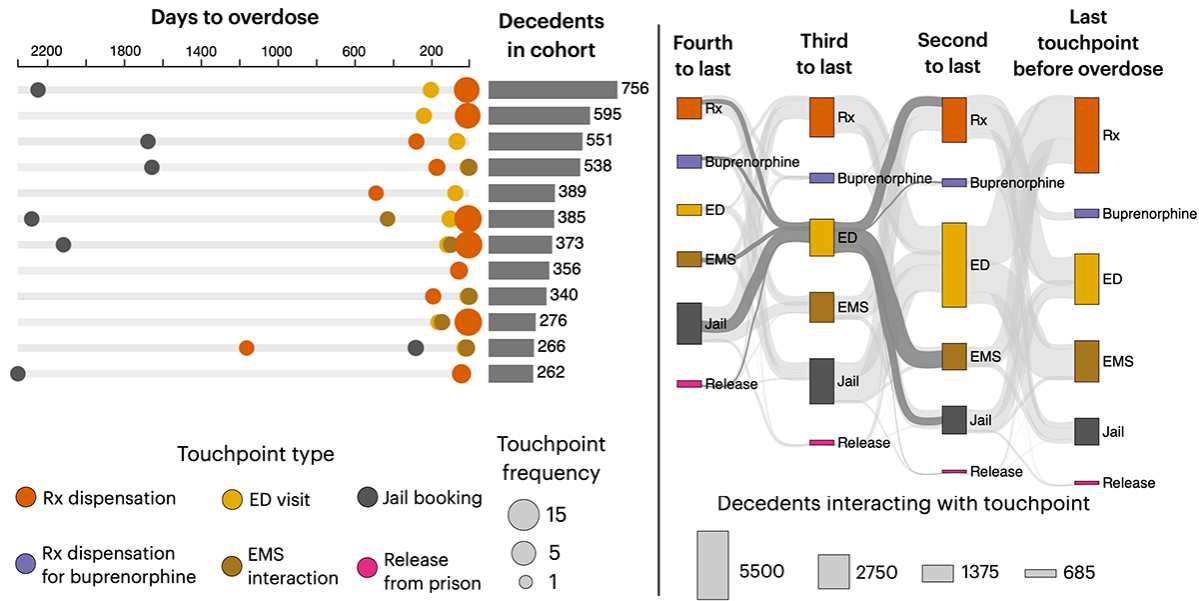
A total of 2 initial visual representations of touchpoints in Indiana (aggregate data from 2015 to 2022). On the left, a timeline-based visualization illustrates the cohorts of decedents with distinct sequences of touchpoints. The visualization depicts the average number of days to fatal overdose (circle position) and frequency of interaction with a touchpoint (circle diameter). For example, the first row shows 756 individuals who experienced a jail booking approximately 6 years before overdose, followed by a sequence of emergency department (ED) visits and medical prescription (Rx) dispensations, the last of which typically occurred approximately 200 and 90 days before overdose, respectively. A Sankey diagram (right) displays the temporal ordering of (up to 4) touchpoints but without showing durations. EMS: emergency medical services.

Our initial visualization focused on timelines, illustrating cohorts of decedents who exhibited similar patterns of touchpoints before overdosing. For example, in Figure 1 (left), each row represents hundreds of decedents who exhibited a similar touchpoint sequence (eg, jail booking followed by one or more ED visits and then a series of prescriptions). This particular visualization was inspired by OFR teams’ use of timelines to represent the histories of individuals discussed during case reviews. However, these initial visualizations received mixed reviews from the expert panel—while they were considered appealing and “interesting,” the focus on cohorts was seen as providing excessive detail for OFRs. This feedback was used to revise the visualizations and develop a final dashboard.

### Final Dashboard

**Figure 2:**
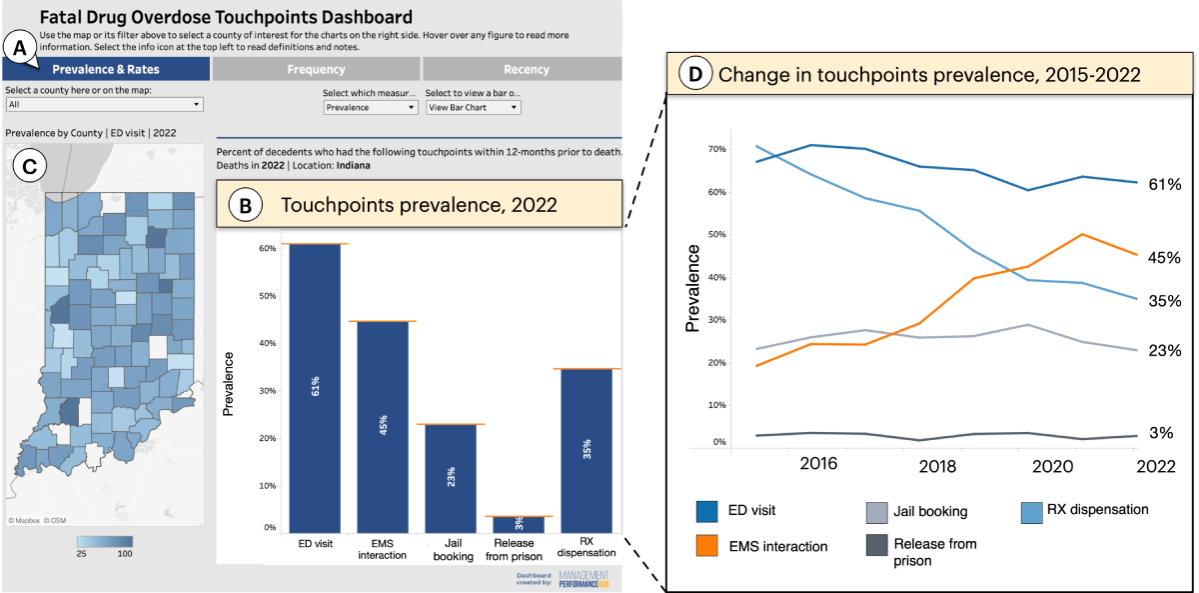
The final dashboard showing overall touchpoints prevalence in Indiana. (A) Buttons enable the user to switch among 4 measures: prevalence, rates, frequency, and recency of touchpoints. (B) The selected measure is visualized here as a bar chart comparing touchpoint prevalence (ie, the percentage of decedents who used each of the 5 touchpoints). (C) A map shows touchpoint prevalence (in this case for emergency department [ED] visits) by county, where darker shades of blue indicate higher prevalence. (D) As an alternative to the bar chart, a line graph allows users to observe how the prevalence of the touchpoints changes from year to year. EMS: emergency medical services; Rx: medical prescription.

The dashboard consists of 3 primary displays (Figure 2A) showing the prevalence and rates, frequency, and recency for the 5 touchpoints. The dashboard can be accessed at the MPH website [45].

#### Prevalence and Rates

**Figure 3:**
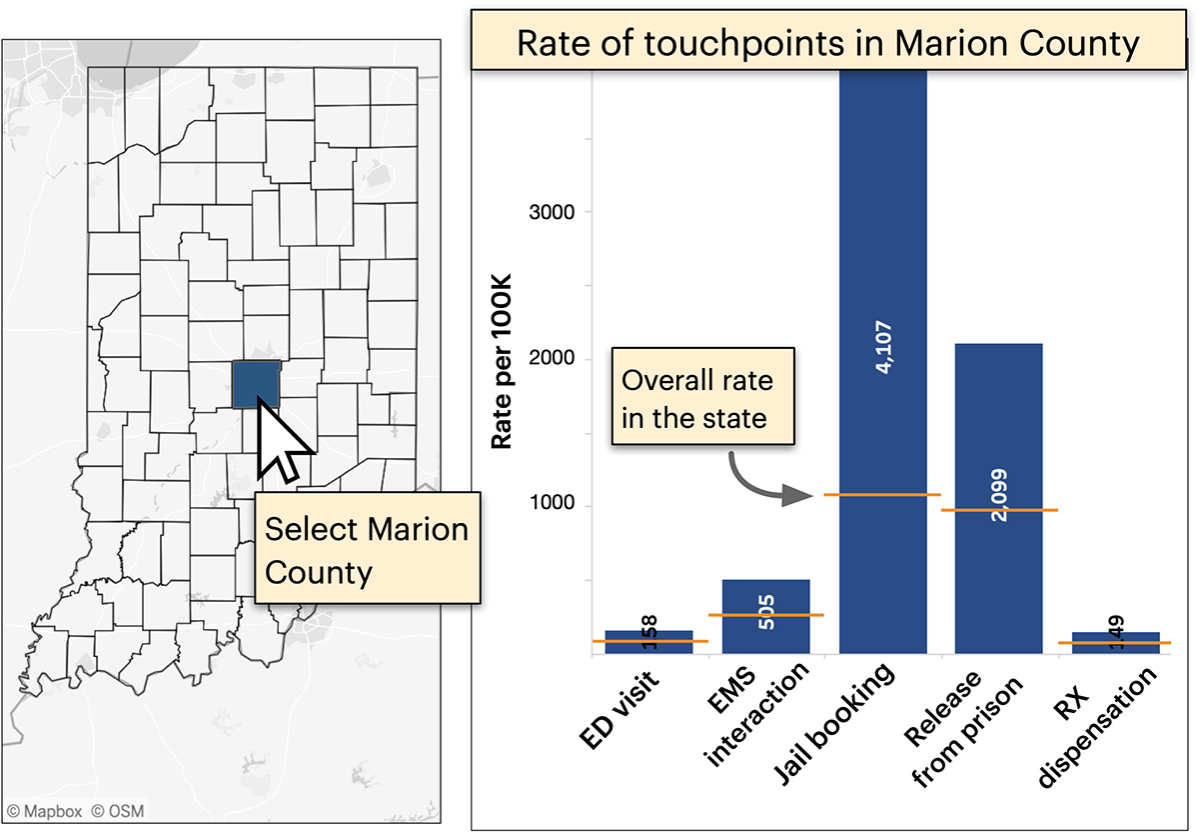
Rates showing the fraction of individuals who experienced a fatal overdose for every 100,000 people who use a touchpoint (right). A map allows the user to filter the data by county, in this example, to show rates for Marion County only. Orange dash marks depict the state average for context. ED: emergency department; EMS: emergency medical services; Rx: medical prescription.

By default, the dashboard displays touchpoint *prevalence*, depicting the percentage of decedents who used various touchpoints in the 12 months preceding overdose. For instance, in 2022, the highest-prevalence touchpoint was the ED, with 61% of individuals who overdosed in Indiana having visited the ED within a year before dying (Figure 2B). The user can also see the change in prevalence over time. For example, the data show that the prevalence of ED visits decreased over time, whereas the proportion of decedents who use EMS increased >2 times between 2015 and 2022 (Figure 2D). In addition to showing state levels, the dashboard can break down the data by county. For instance, the user can see the prevalence of ED visits in different counties on a map (Figure 2C). Notably, the map shows 4 counties in which practically all decedents had visited the ED a year before their overdose. The map can also be used to filter the bar or line graph displays. For example, clicking on Marion County, the most populous in Indiana, updates the display to show statistics for Marion only (Figure 3).

In addition to prevalence, the dashboard visualizes the *rate* of touchpoints among decedents. These rates depict the number of fatal overdose cases per 100,000 individuals who typically use services such as the ED. Unlike prevalence, which indicates the likelihood of a decedent using a touchpoint, rates reveal the probability of a fatal overdose after using 1 of the 5 legal or medical touchpoints included in the dashboard. Both measures are important for resource allocation—while prevalence helps users identify touchpoints with the broadest reach, rates can reveal more “efficient” touchpoints for targeted interventions. For example, consider jail bookings and releases from prison (Figure 3 [right]), which exhibit the highest rates among touchpoints in Marion County. This offers a high-specificity opportunity to focus on individuals at a greater risk of overdosing despite these touchpoints exhibiting relatively moderate to low prevalence at the state level (23% and 3%, respectively, as depicted in Figure 2 [left]).

#### Touchpoint Frequency

**Figure 4:**
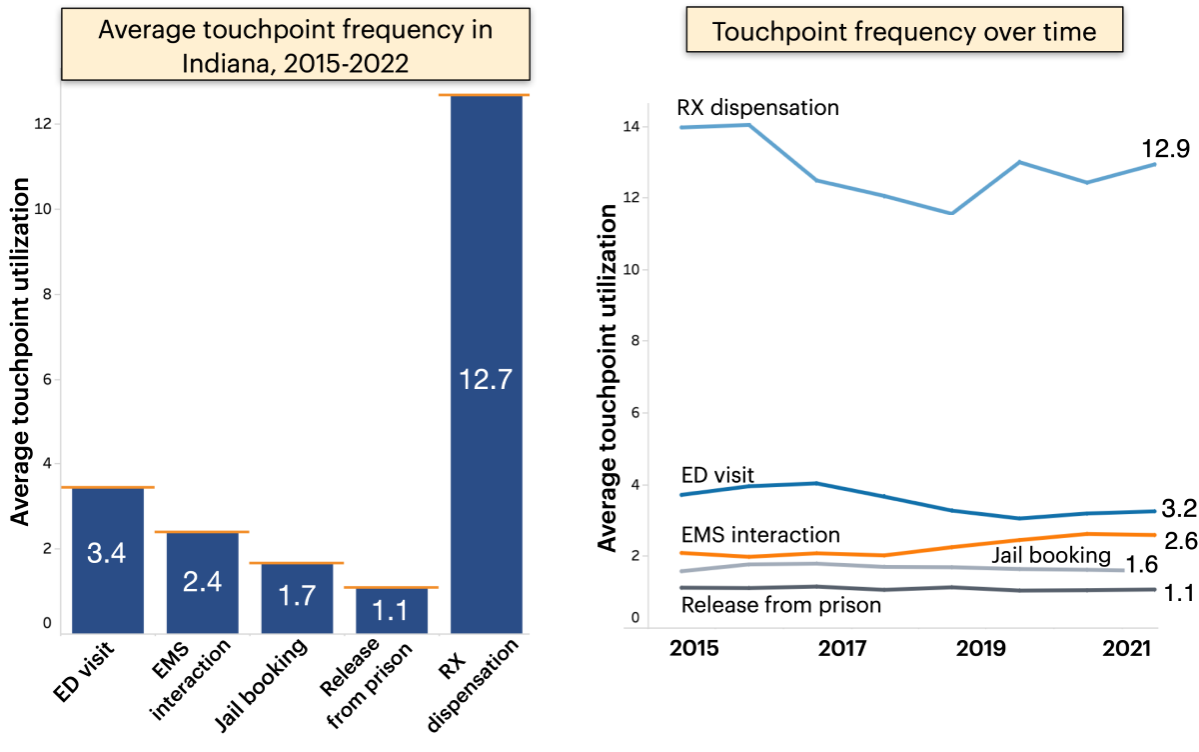
Average number of interactions with the 5 touchpoints from 2015 to 2022 (left) alongside a year-by-year breakdown. ED: emergency department; EMS: emergency medical services; Rx: medical prescription.

The second display summarizes the average number of interactions a decedent had with a touchpoint in the year preceding their overdose (Figure 4). Notably, the most frequently used touchpoint in the state is medical prescription (Rx) dispensation for controlled substances, such as an opioid analgesic (12.7 events on average at the time of writing). The user can also see how this frequency changes yearly (Figure 4 [right]). The line graph shows relatively stable use for ED, EMS, and criminal justice services, with the average number of Rx dispensations trending down slightly.

#### Recency

**Figure 5:**
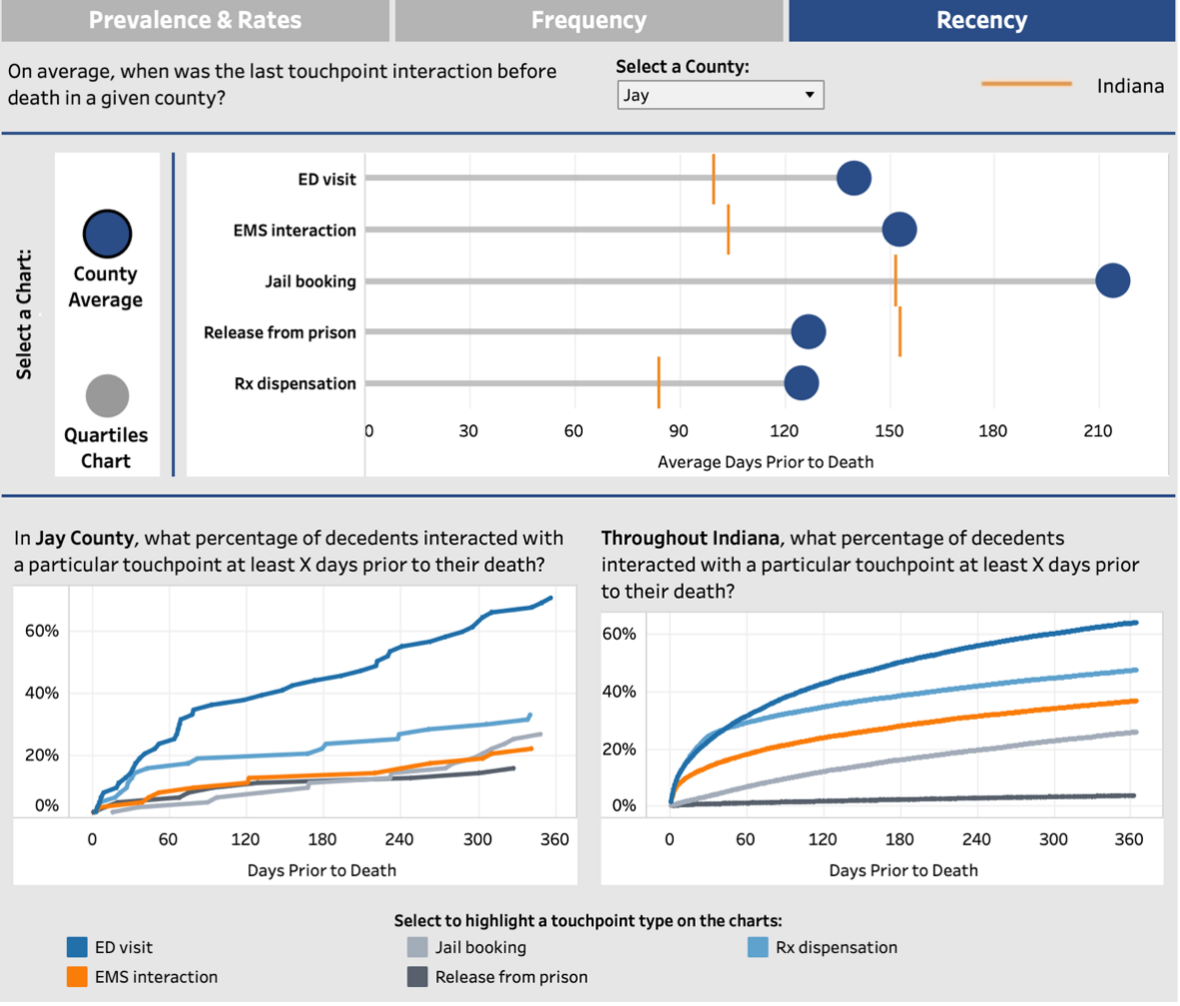
The average time gap between the final interaction and overdose events across different touchpoints (top). The lower section comprises 2 charts demonstrating the cumulative reach of touchpoints at varying time intervals, comparing the selected county (bottom left) with the state average (bottom right). ED: emergency department; EMS: emergency medical services; Rx: medical prescription.

The timing of interaction with services was identified as a key factor for OFRs. Accordingly, the *recency* display illustrates the typical time intervals between final touchpoints and overdose events (Figure 5). The top features a “lollipop” chart depicting the number of days on average between the most recent interaction and the overdose (Figure 5 [top]). In this example, jail bookings in Jay County (selectable by the user) occur approximately 210 days on average before a fatal overdose compared to approximately 150 days for the entirety of Indiana. Conversely, releases from prison tend to happen approximately 120 days before the overdose, closer relative to the state average. The bottom visualizations show a curve for each touchpoint representing the cumulative percentage of individuals who could have been engaged at various time points relative to their time of death. In this case, approximately 27% of decedents in Jay County could have been engaged through an Rx dispensation touchpoint 30 days before an overdose.

#### Aiding Data Interpretation

**Figure 6:**
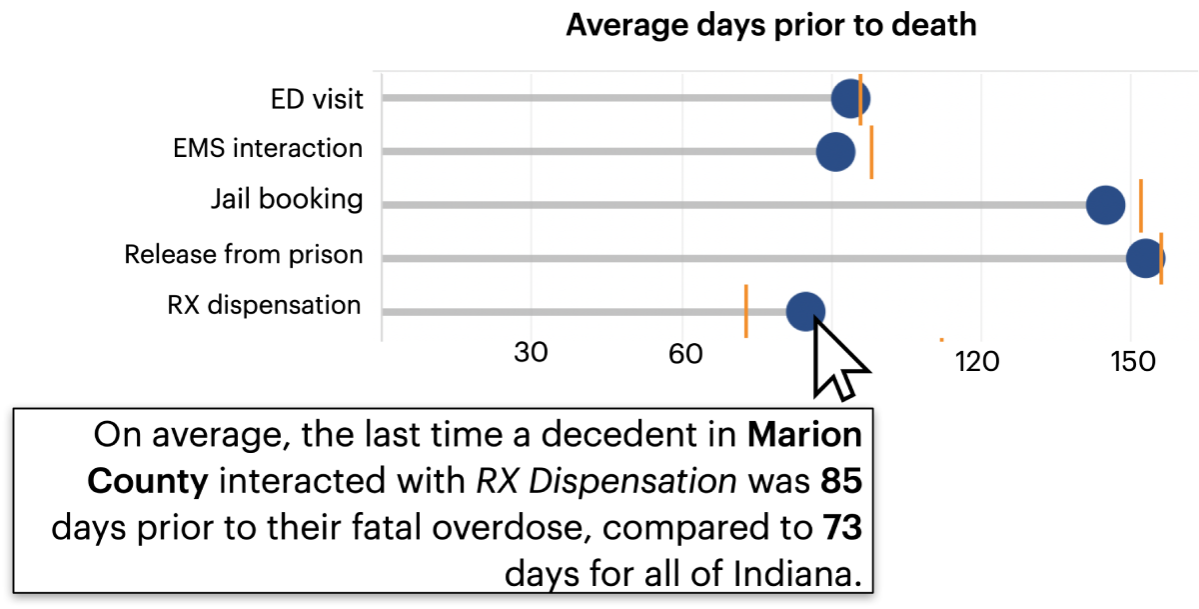
Tooltips appear throughout the dashboard to promote accurate data interpretation. ED: emergency department; EMS: emergency medical services; Rx: medical prescription.

One concern that emerged during the focus groups regarded OFR teams’ ability to interpret population-level statistics. To aid users in making sense of these data, the dashboard provides tooltips in the form of short text annotations that explain the interpretation of each visualization. For instance, in the recency chart, the text clarifies that the points depict the average number of days between a touchpoint and an overdose event (Figure 6).

### Initial Evaluation Results

We invited 3 OFR experts to review and provide feedback on the dashboard. They commented on features they thought were beneficial. They also provided suggestions on how to ensure dashboard integration into OFR practices. One of the notable strengths of the dashboard was its comprehensive data coverage, a feature that was highly appreciated by all participants. They specifically praised the breakdown of touchpoints on a county basis, a level of granularity that is often lacking in existing dashboards. The inclusion of small counties, the data on which can be especially difficult to obtain, was recognized as a significant advantage. Participants also appreciated the ability to compare different counties through the map, along with the ability to juxtapose county-specific data against state averages.

Among the various visualizations, the recency chart (referred to as the “timeline”) stood out for its depiction of events leading up to overdoses. Participants thought that these temporal data, which can be difficult to obtain at the population level, can help in tailoring interventions:

It is interesting to see this [chart], and to know what can be done with data. We can check the timeline and help implement a strategy. Through these strategies, we can outline short, medium, and long-term goals.

In thinking about how the dashboard might complement existing OFR practices, participants highlighted its usefulness in guiding case selection for review and helping OFRs build a representative case profile. One participant specifically noted the potential of the dashboard in conducting “community data review” to explore “what is going on in my community.” Moreover, the dashboard’s availability on a publicly accessible URL was lauded as “a wonderful resource,” extending its value to audiences beyond OFRs. The discussion opened the door for offering some form of training or educational support to OFR members, equipping review teams with skills to interpret quantitative data. One participant suggested the addition of a “demo video to help interpret and apply the data.” Another suggested the need to specifically focus on OFR facilitators as crucial personnel for communicating data insights to review teams:

I don’t think they [members of the review teams], will be able to fully understand the data, so training the facilitator will be key.

## Discussion

### Principal Findings

OFR teams are proliferating in the United States, becoming an important public health tool to combat the drug overdose crisis. Traditional fatality reviews, often limited to a few cases, do not fully capture the broader overdose trends, especially in communities with numerous drug-related fatalities. This research aimed to enhance OFR data use by addressing data access barriers, identifying information needs, and creating actionable visualizations of population-level overdose data.

Our findings shed light on challenges that OFR teams face in accessing timely data, frequently impeded by legal constraints. When available, these data can often be inconsistent, for example, in the coding of events and classification of services. Despite these challenges, OFR teams seemed keen on incorporating a wider range of data into their review to better understand the factors contributing to overdose risks in their communities. Notably, the expert panel highlighted several key touchpoints, including incarcerations, interactions with substance treatment services, and visits to medical facilities such as EDs.

Some of these touchpoints have been previously recognized as opportunities for delivering prevention services [25,46,47]. For example, the time window following a prison release has been identified as a particularly critical and risky period, making this touchpoint a highly specific and valuable opportunity for administering prevention services [48-50]. However, effectively sharing these data insights with OFRs remains a challenge. Our findings suggest that a dashboard linking state administrative and mortality data could effectively provide local OFRs with insights on the timing and distribution of touchpoints. To explore this potential, we partnered with the Indiana state government and developed a dashboard that collates and visualizes data on 5 touchpoints at the county level, enabling OFR teams to see statistics and patterns on events that *precede* fatal overdoses in their community. To our knowledge, this is the first system to automatically analyze touchpoint characteristics and offer (near) real-time visualizations of their prevalence, frequency, and timing tailored to the local scale of OFR teams. In designing the dashboard, we specifically focused on this user group and prioritized actionable data that shed light on local prevention opportunities. The developed touchpoint dashboard stands in contrast to earlier dashboards for opioid prescription and overdose data, which are meant for the public or nonspecified stakeholders.

Our OFR expert panel suggested that one of the most crucial pieces of information is the timing of touchpoints—specifically, the average duration between an individual’s last encounter and their overdose. The dashboard prominently features these data in a lollipop chart comparing the *recency* of various touchpoints. In addition, we incorporated displays of touchpoint prevalence and rates, providing insights into the reach of touchpoints and the specificity they afford for targeting individuals who are at high risk of overdose. The dashboard purposely uses familiar visualizations, including bar and line graphs and choropleth maps, to appeal to review teams who may be novice visualization users [51]. Importantly, the dashboard breaks down these statistics at the county level, aligning with how OFRs are organized in Indiana. By visualizing data “close to home,” we aimed to improve the actionability of the dashboard [52]. However, users can easily compare county data to state averages or those of other similar counties.

Our initial evaluations show promise for the dashboard’s usefulness. However, successfully integrating the dashboard into OFR practices will likely require training for OFR members, many of whom lack expertise in data analysis—a point that was notably underscored by the expert panel. In particular, teams may need educational support in how to interpret population-level features, such as the difference between the prevalence and rates of touchpoints. Regular meetings with OFR users could also help uncover usability issues and gauge dashboard adoption by review teams.

While the dashboard offers detailed insights into community touchpoints, it omits data on social determinants such as race, educational level, and access to housing and harm reduction services. These factors can be important for understanding overdose risks, as per our expert panel and research findings [53,54]. Future versions of the dashboard could incorporate local statistics on these risk factors. Furthermore, it is possible to expand the current list of touchpoints to include specific events associated with social determinants, such as loss of housing or employment. These additional touchpoints could offer further intervention avenues to disrupt pathways from marginalization to overdose [55]. Another limitation is that, while the dashboard includes critical touchpoints such as ED and EMS encounters, these events currently lack classification. Adding a breakdown of these touchpoints, for example, by distinguishing between substance-related versus other EMS encounters, could enable OFR teams to further tailor their recommendations.

The experts interviewed also sought demographic breakdowns of touchpoint data, in part to ensure that diverse populations would benefit from interventions at touchpoints. Unfortunately, this feature was not included in the current dashboard due to reidentification risks, particularly in rural areas that have fewer overdoses. In the future, the dashboard could be modified to provide a demographic breakdown of touchpoints at the aggregate (eg, state) level to substantially decrease the risk of reidentification instead of withholding these data altogether. To further protect individual confidentiality, which was a key concern of our expert panel, the dashboard presents data as percentages (eg, the proportion of decedents who were released from prison within a year before their overdose) and rates. Withholding the actual counts for events helps prevent the inference of individual identity in places where those counts are low. The dashboard provides a visual warning for statistics based on <20 cases, cautioning users against drawing strong conclusions from small samples. Future work could use more advanced privacy-preserving techniques [56,57], thus allowing for the display of a wider range of attributes without jeopardizing anonymity.

Although our dashboard is specific to Indiana, we believe that the approach could be adapted for other US states and localities. This expansion requires access to overdose mortality records that can be algorithmically cross-referenced with other administrative data sets. Many states already have data infrastructure for such linked analyses [58,59]. We estimate that the development and maintenance of the dashboard over 2 years will require approximately 350 personnel hours assuming the availability of data. The prevalence of overdose dashboards [39,60] indicates both the technical feasibility of creating such tools and the interest in them from the public health community. Our research demonstrates that dashboards can go beyond surveillance to directly visualize actionable prevention opportunities.

### Conclusions

OFRs can play a crucial public health role in understanding overdose cases and recommending prevention strategies. This study explored the potential for enhancing these reviews with population-level data for broader, quantitative insights into risk factors. Following a user-centered design process, we developed a dashboard that tracks and visualizes decedents’ encounters with medical and justice systems at the county level. Although initially designed for Indiana, the dashboard can be adapted to other localities, leveraging administrative and mortality data typically collected by local governments. Preliminary evaluation shows the potential utility of the dashboard for analysis and case selection but emphasizes the need for training OFR members in data interpretation and decision-making.

## Abbreviations

ED: emergency department
EMS: emergency medical services
MPH: Management Performance Hub
OFR: Overdose Fatality Review
Rx: medical prescription

## Data Availability

The video recording data sets generated during and analyzed during this study are not publicly available to protect participants’ confidentiality but are available from the corresponding author on reasonable request. The aggregate touchpoints data sets are available in the Management Performance Hub (MPH) data repository [61].
